# Post-bariatric hypoglycaemia diagnosed during pregnancy

**DOI:** 10.1530/EDM-23-0010

**Published:** 2023-10-03

**Authors:** Dave Duggan, Cinthia Minatel Riguetto

**Affiliations:** 1 Waikato Regional Diabetes Service, Te Whatu Ora Health New Zealand, Hamilton, New Zealand

**Keywords:** Adult, Female, Other, New Zealand, Pancreas, Diabetes, Unique/unexpected symptoms or presentations of a disease, October, 2023

## Abstract

**Summary:**

There is a scarcity of literature relating to post-bariatric hypoglycaemia (PBH) in pregnancy. Recurrent hyperglycaemia and hypoglycaemia can have significant consequences for both the mother and the developing fetus. We describe a case of a young pregnant woman who was diagnosed with symptomatic PBH in the second trimester of pregnancy using continuous glucose monitoring (CGM) 3 years after Roux-en-Y gastric bypass (RYGB) surgery. Instigating a low glycaemic index and complex carbohydrate diet significantly improved the patient’s glycaemic excursions. Given that this condition is likely underdiagnosed as a complication of RYGB surgery, a greater awareness of this complication is needed. Patients should be adequately consented pre-operatively for this relatively frequent late surgical complication to enable patients to identify symptoms of this condition at an early stage and seek medical treatment.

**Learning points:**

- PBH is an important diagnosis in patients post-RYGB surgery, particularly in women of childbearing age when consequences of both hyperglycaemia and hypoglycaemia during pregnancy can adversely affect both mother and the fetus.
- Adverse outcomes of recurrent hypoglycaemia to the fetus can include small for gestational age, intrauterine growth restriction and possible impairment of beta cell function.
- Providing adequate carbohydrate intake to allow growth of the fetus during pregnancy while also attempting to resolve both hyperglycaemia and hypoglycaemia associated with PBH by reducing the intake of simple carbohydrates and high glycaemic index foods can prove challenging.
- Patients should be adequately consented for late complications of RYGB surgery such as PBH in order to allow early recognition of symptoms and enable prompt treatment.

## Background

Roux-en-Y gastric bypass (RYGB) can be an effective tool in achieving sustainable weight loss in the obese population with long-term improvements in associated co-morbidities and health outcomes. PBH is likely under-recognised as a complication of such surgery. With the increased use of continuous glucose monitoring (CGM), this has allowed real-time viewing of glucose patterns relating to meals and has facilitated the identification of more patients with this condition. An increased rate of accidental death has been reported in patients who have undergone RYGB, and it is speculated that this could result from severe undetected hypoglycaemia (1). It is therefore paramount to make this diagnosis promptly in order to commence treatment and reduce associated glycaemic excursions. We describe a case of symptomatic PBH diagnosed in a pregnant woman who was unaware of this late complication of RYGB and who’s symptoms had likely been present on and off for at least 2 years.

## Case presentation

A 32-year-old Māori pregnant woman, G1P0, with a 5-year history of type 2 diabetes and a pre-pregnancy glycosylated haemoglobin of 45 mmol/mmol presented for management of her diabetes. Anti-GAD and anti-IA2 antibodies were negative at the beginning of pregnancy, and there was no evidence of microvascular diabetes complications. She also had a past medical history of obstructive sleep apnoea, non-alcoholic fatty liver disease and vitamin B12 deficiency.

The patient was initially diagnosed with type 2 diabetes after identification of an elevated glycosylated haemoglobin of 59 mmol/mmol in 2017. She required Lantus 10 units daily as well as gliclazide and metformin to control her blood glucose levels at the time. Two years after this diagnosis, she underwent a laparoscopic RYGB surgery for obesity. Her weight dropped from 130 kilos to 97 kilos post-surgery. The patient’s diabetes was subsequently able to be controlled by diet alone.

At 11 weeks gestation, night-time isophane insulin was commenced for elevated fasting morning glucose levels. Occasional significantly elevated 2-h postprandial glucose levels, which reached up to 11 mmol/L were also noted at this time. However, most glucose readings were within the target range for pregnancy of 4.0–6.5 mmol/L. At 14 weeks gestation, she was reviewed by her Diabetes in Pregnancy Dietician and noted to be frequently omitting lunch and dinner due to work commitments and only eating approximately 90 g of carbohydrates daily. She was advised to take more regular breaks and increase her carbohydrate intake, which she implemented. The patient subsequently confirmed diaphoresis, palpitation and light headedness, particularly after eating her lunch and evening meals, with symptoms relieved by drinking juice or eating chocolate. She retrospectively reported intermittent ongoing similar symptoms after simple carbohydrate rich meals from shortly after her RYGB.

## Investigation

**Figure 1:**
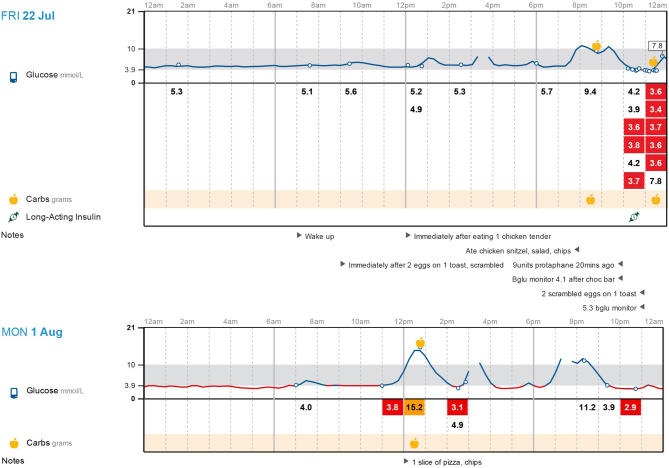
Patient’s food diary while using CGM at 18 weeks gestation showing high glycaemic index foods to be the main precipitants of postprandial hypoglycaemia. CGM, continuous glucose monitoring.

**Figure 2:**
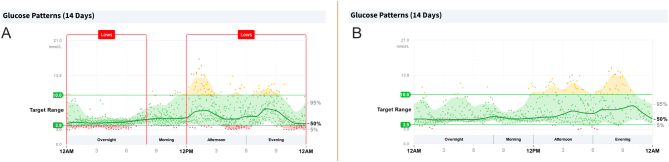
(A) Colour-enhanced glucose pattern over the 14-day period before making dietary changes at 18 weeks gestation. The graph demonstrates significant glycaemic variability with elevated interstitial glucose levels post-lunch and -dinner, with a later drop. (B) Colour-enhanced glucose pattern over the 14-day period after making dietary changes at 23 weeks gestation. The graph demonstrates significant improvement in postprandial hypoglycaemic episodes.

At 18 weeks gestation, she had a 2-week trial of CGM. Data analysis from CGM showed 13% of interstitial glucose (< 3.9 mmol/L) with readings as low as 2.8 mmol/L and 7% >10 mmol/L, as well as high glucose variability of 38.3%. The patient kept a thorough food diary while on CGM, allowing the revelation of meals with high glycaemic index foods and simple carbohydrates to be the main precipitants of her significant mealtime glucose variability (Fig. 1). Hyperglycaemic spikes were noted immediately after eating lunch and dinner with hypoglycaemic episodes present 2 hlater as shown in her ambulatory colour-enhanced glucose pattern in Fig. 2A. The patient also confirmed 2-h post-meal hypoglycaemia using blood glucose monitoring with readings between 3.0 mmol/L and 3.8 mmol/L. Her nocte isophane insulin was reduced at the time due to mild overnight hypoglycaemia with good effect.

An oral glucose tolerance test or a mixed meal tolerance test was not performed as these provocative tests may cause uncomfortable symptoms and dangerous hypoglycaemia in patients who have had prior gastric bypass surgery which is important to avoid in pregnancy (2).

## Treatment

Having been made aware of her diagnosis of PBH, the patient relayed that she had assumed her ongoing symptoms post-meals remained part of her early dumping syndrome despite it being 3 years since her surgery. She was unaware that her symptoms had likely transitioned at some point after the first year to those of PBH. The patient’s Diabetes in Pregnancy Dietician subsequently implemented a low glycaemic index, complex carbohydrate and high fibre diet. This involved three main meals of 45 g of carbohydrates in addition to three snacks of 15 g of carbohydrates to meet daily carbohydrate requirements during pregnancy.

## Outcome and follow-up

Having been empowered by the explanation of her symptoms and diagnosis, the patient was able to make a conscious effort to avoid simple carbohydrates, high glycaemic index and high-fat foods with good effect. This successfully resolved most mealtime hyperglycaemic and hypoglycaemic episodes. This was confirmed by reported resolution of the patient’s symptoms as well as a new CGM trial at 23 weeks gestation verifying both 1- and 2-h postprandial capillary glucose levels within the target range (Fig. 2B). This negated the need for medication use to control symptoms, and the pregnancy progressed without any significant complications to the mother or fetus. She required a C-section for fetal distress at 38 weeks and delivered a healthy newborn weighing 2.8 kilos at birth. There was no newborn hypoglycaemia post-delivery.

## Discussion

PBH is most common after RYGB surgery, occurring in both diabetes and non-diabetes patients. There is a large discrepancy in the prevalence of this condition due to different diagnostic approaches and no consistent definition of the condition. In recent years, CGM has proven a useful tool for detecting and monitoring hypoglycaemia post-RYGB, demonstrating asymptomatic hypoglycaemia rates of greater than 30% (3). Pregnant women post-RYGB have also been shown to have more frequent hypoglycaemia and hyperglycaemia during pregnancy using CGM than body mass index-matched pregnant controls who have not had this surgery (4). CGM has its limitations with occasional low readings reported in the first 24–48 hof its use, decreased accuracy in the hypoglycaemic range and variability of readings due to oedema in late pregnancy.

Hypoglycaemia in pregnancy carries significant risks to both the mother and fetus. Fetal complications from hypoglycaemia include small for gestational age, intrauterine growth restriction and possible impairment of beta cell function (5). Recurrent maternal hypoglycaemia may impair cognition, reduce the quality of life and increase all-cause mortality (6). In addition, hyperglycaemia during pregnancy is associated with a greater risk of miscarriage, pre-eclampsia, macrosomia and overall perinatal mortality (7).

The pathophysiology of PBH likely involves alterations in multiple hormonal and glycaemic patterns, including elevated postprandial plasma glucose with subsequent exaggerated postprandial insulin and glucagon-like peptide 1 (GLP-1) levels after an oral meal. Hypersecretion of GLP-1 and glucose-dependent insulinotropic polypeptide may induce beta cell expansion by inducing transcription factors such as pancreatic and duodenal homeobox-1 protein (8). Post-surgical nesidioblastosis has also been proposed to play a role in this condition. Pancreatic resection to treat nesidioblastosis has been used for refractory cases of PBH but has unproven benefits and is not recommended.

An oral glucose tolerance test should be avoided as up to 10% of patients with a healthy pancreas will drop their blood glucose to <2.8 mmol/L with the additional risks of severe post-absorptive hypoglycaemia and dumping syndrome in PBH (2). A mixed meal tolerance test containing carbohydrates, protein and lipids is a more physiologic provocative test that can be used to confirm the diagnosis.

One review article by Kirby *et al.* recommended 30 g of carbohydrates three times daily plus 15–30 g of snacks as an effective tool to prevent PBH symptoms. However, this is not feasible in pregnancy as nutritional requirements warrant, at a minimum, 175 g of carbohydrates daily (9). Although patients who are refractory to dietary modifications can be treated with medications, some of these medications are contraindicated in pregnancy. The potassium channel activator diazoxide may cause developmental toxicity in the third trimester and should be avoided (10). There is anecdotal evidence that GLP-1 receptor agonists may be helpful in patients with PBH, possibly due to reduced gastric emptying, but have not been studied in human pregnancy and therefore are contraindicated (10). Octreotide crosses the placenta and may cause issues with both maternal hyperglycaemia and hypoglycaemia as well as bile stone formation but is likely of low pregnancy toxicity risk (10). Nifedipine, a calcium channel blocker, can be used safely in pregnancy to help reduce insulin secretion in PBH (10). The alpha-glucosidase inhibitor, acarbose, has low systemic bioavailability and has been safely used in pregnancy; however, it may cause significant gastrointestinal side effects (10).

PBH can complicate pregnancy, particularly as more carbohydrates are required to sustain the mother and allow adequate fetal growth. There is an ever-growing number of people seeking bariatric surgery at a young age, a population that often do not adhere to postoperative behavioural recommendations. It is therefore imperative that patients, in particular women of childbearing age, are consented for this late complication prior to RYGB surgery. Awareness of the symptoms of this condition and prompt reporting of these to a medical professional can allow early diagnosis and prevent the subsequent risks of hyperglycaemia and hypoglycaemia that have potential consequences for both mother and fetus.

## Declaration of interest

There is no conflict of interest that could be perceived as prejudicing the impartiality of the research reported.

## Funding

This research did not receive any specific grant from any funding agency in the public, commercial or not-for-profit sector.

## Patient consent

Written informed consent for publication of clinical details and/or clinical images was obtained from the patient.

## Patient’s perspective

Ever since having the gastric bypass, I have noticed feeling hot, clammy, short of breath and tired and could feel my heart beating quickly straight after a meal that contained any carbohydrates. This would resolve after half an hour if I just sat and waited it out. This meant that I started taking lunch breaks alone without my colleagues, as they would often ask me if I was feeling sick at the end of the meal, which I found embarrassing. It also meant that if I was pushed for time, I wouldn’t have a quick bite to eat for lunch as I would feel unwell when I went back to work. So, I adjusted to having lunch alone and would make sure I had plenty of time to recover from my meal. Around 2 h after a meal, I typically felt very tired and would almost fall asleep, so would try to walk around the office to stay awake. Only occasionally would I start to feel shaky, and so would have a hot chocolate to resolve this. I thought these symptoms were a continuation of early dumping syndrome and so was not particularly concerned and didn’t seek out further medical advice about this. My sister, who doesn’t have a diagnosis of diabetes but had a gastric bypass a year before me, also experiences the exact same symptoms. I cannot recall being told about late dumping syndrome by my gastric bypass surgeon, but it is possible this was discussed. PBH was only picked up when I became pregnant and had to start monitoring my blood glucose levels because I had pre-existing type 2 diabetes. Changing to a low glycaemic diet has been challenging and continues to be a work in progress, as difficulties maintaining a healthy diet were what started me on the journey of having a bypass and having diabetes in the first place. Fortunately, I am receiving excellent support from the diabetes team and dietician, and my husband is supportive of changing to lower glycaemic index products in our home, which is helping me on my way.

## Author contribution statement

Supervised by CMR. Patient was under the care of CMR. Diagnosis was made by DD with the guidance of CMR. Report written by DD and CMR. All authors read and approved the final manuscript.
